# The Greening of Anthocyanins: Eco-Friendly Techniques for Their Recovery from Agri-Food By-Products

**DOI:** 10.3390/antiox11112169

**Published:** 2022-11-01

**Authors:** Mariacaterina Lianza, Lorenzo Marincich, Fabiana Antognoni

**Affiliations:** Department for Life Quality Studies, Rimini Campus, University of Bologna, Corso d’Augusto 237, 47921 Rimini, Italy

**Keywords:** agri-food waste, anthocyanins, by-products, green solvents, green extraction techniques, plant metabolite recovery

## Abstract

In recent years, several steps forward have been made toward a more sustainable approach for the extraction of bioactive compounds from plant materials based on the application of green extraction principles. It is currently recognized that waste and by-products deriving from agriculture and food industries still contain a wide array of high value-added substances, which can be re-used to obtain new products with various applications in the food, supplement, pharmaceutical, and cosmetic industries. Anthocyanins are a class of these valuable metabolites; they confer the red, violet, and blue color to fruits and vegetables, and scientific evidence has accumulated over the last few decades to support their beneficial effects on human health, in great part deriving from their powerful antioxidant capacity. This review provides a general overview of the most recent green procedures that have been applied for the recovery of anthocyanins from plant-derived wastes and by-products. The most widely used green solvents and the main sustainable techniques utilized for recovering this class of flavonoids from various matrices are discussed, together with the variables that mainly impact the extraction yield.

## 1. Introduction

To face the current challenges, i.e., climate change, biodiversity loss, and increasing environmental pollution, the need to improve relationships between humans and ecosystems has arisen. To this purpose, the EU environmental policy and legislation have set as a priority the re-use and recycling of wastes, the reduction in harmful chemicals, and the use of new and more environment-friendly compounds, which can meet both technological and economic demands [1].

Over the last few decades, plant-derived by-products have been recognized as valuable sources of bioactive compounds. Their sustainable use to obtain new products has recently emerged as a useful tool for exploiting several classes of plant-derived compounds for different applications, such as for food, supplement, pharmaceutical, and cosmetic industries, among others [2,3].

One of the most interesting groups of plant specialized metabolites is represented by anthocyanins, water-soluble compounds that confer red, violet, and blue color to plant organs [4]. From a chemical point of view, anthocyanins are sugar conjugates of anthocyanidins (aglycones), a subclass of flavonoids with a positive charge at the oxygen atom of the C-ring, also called a flavylium ion, that confers an ionic nature. The most common aglycones known are cyanidin, peonidin, pelargonidin, malvidin, delphinidin, and petunidin, which differ from each other in their position in hydroxyl and methoxyl groups [5]. As regards sugar moieties, glucose, rutinose, arabinose, and galactose are the most prevalent ones. Anthocyanins also form conjugates with hydroxycinnamates and organic acids, such as malic and acetic acids.

Major sources of anthocyanins in the diet are fruits and some dark-colored vegetables, such as blueberries (*Vaccinium corymbosum* L.), bilberries (*Vaccinium myrtillus* L.), cranberries (*Vaccinium macrocarpon* Aiton), red grapes (*Vitis vinifera* L.), pomegranates (*Punica granatum* L.), eggplants (*Solanum melongena* L.), and red onion (*Allium cepa* L.). Recently, there has been a growing interest in some Amazonian fruits rich in anthocyanins, which are considered superfruits due to their functional properties, such as açai (*Euterpe oleracea* Mart.) [6] and Jabuticaba (*Plinia cauliflora* Mart. Kausel) berries. The processing of these fruits and vegetables to produce juices, jams, energy drinks, and fermented distilled liquors generates high amounts of by-products, such as peels, seeds, and pomace, which are exploitable to obtain new products with market value.

Anthocyanins have a wide array of applications: they can be re-used as colorants in food and drinks, representing a safer alternative to synthetic dyes [7], as food preservatives, thanks to their antimicrobial activity against foodborne pathogens [8], and as a nutraceutical and dietary supplements, given their well-recognized beneficial effects on human health [9,10,11], which are more or less associated with their potent antioxidant properties.

The extraction and separation of bioactive compounds with conventional organic solvents can have a negative environmental impact, since most of them derive from petroleum [12] and are volatile, inflammable, and toxic. Thus, the first step in the development of green techniques is the use of green solvents. They are defined as solvents that fully meet safety, health, and environmental requirements, the latter in terms of both direct and indirect impact, i.e., high biodegradability, low vapor pressure, lower air emissions due to incineration [13,14], and resource use. For a technique to be truly defined as green, special requirements are needed, such as the use of low solvent volumes, a short time frame, and a low number of steps needed to obtain the extract, the latter two being the parameters mainly impacting energy costs. Moreover, a green technique should minimize the production of waste, hazardous substances, and pollution [15].

Based on this background, this review aims to provide an overview of the green techniques that have recently been applied for the extraction of anthocyanins from agri-food by-products, focusing on completely green experimental designs in terms of both procedures and solvents used. A bibliographic survey was carried out, evaluating scientific papers dealing with this aspect. These papers were sourced from the Web of Science database. (https://www.webofscience.com/wos/woscc/basic-search, accessed on 15 July 2022). The keywords used for the search were the following: “(by NEAR products) AND (anthocyanins) AND ((green NEAR extraction) OR (green NEAR solvent))”, excluding reviews as the document type. Using these parameters, 62 articles were found, but only 33 were selected as relevant for the topic since the others did not report truly green methods or the protocols were not specific for anthocyanins. According to our survey, the first paper fitting such features dates to 2013, and 73% of the collected papers were published in the last five years, with the last one (2021) having the highest number of articles.

The results of the survey are summarized in Table 1, which reports the plant species used, the derived by-products and their origin, and for each of them, the green solvents, the green technique applied, the anthocyanin extractive yield, and the type of anthocyanins recovered. The scientific name of the plants was checked on The World Flora Online plant list [16]. A more detailed description of the anthocyanin structures (Figure S1) recovered from the various plant sources, as well as the detection techniques used for their identification, are provided in Table S1 (Supplementary Material).

The green solvents and the techniques more frequently applied for the recovery of anthocyanins from different by-products, together with the variables that mainly impact the extraction procedures for these biomolecules, are here discussed.

## 2. Green Solvents for Anthocyanin Extraction from Agri-Food By-Products

### 2.1. Water

Water is, by far, the greenest solvent, being the safest and the most environment-friendly one. These features, together with the easily accessible infrastructures available for its supply, make water one of the most sustainable solvents. However, care should also be given to properly clean up aqueous wastes generated during the extraction processes, in addition to considering the worldwide water availability in the long term when using this solvent to treat huge masses of waste at an industrial level [48]. Moreover, it should be considered that a high amount of energy is required to remove water from a plant extract. Thus, when performing an extraction with water or a water-containing mixture, it is not strictly necessary to dry the starting plant material, thus offsetting the energy consumption to obtain the final dry extract.

The chemical structure of water, possessing a dipole moment, confers a high hydrogen bonding capacity, which is essential for both water–water and water–biomolecule interactions. Thus, water is suitable for extracting polar compounds, such as anthocyanins. However, increasing the temperature, both the polarizability and the degree of the hydrogen bonding capacity decrease [49], and this allows the solubilization of less polar compounds. Another way to modify the polarity of water is to mix it with miscible solvents (e.g., ethanol, natural deep eutectic solvents, and glycerol), as in the case of several studies cited below.

Water, either pure or mixed with other solvents, emerged as the solvent of choice for the green recovery of anthocyanins in several cases. For the valorization of saffron (*Crocus sativus* L.) by-products, four out of five studies used just water or acidified water [19,21,22,23]. The only one that did not report the use of water applied cold pressing, which is actually a solvent-free technique [20]. In a research carried out by Stelluti et al. [23], pure water, coupled with either ultrasound-assisted extraction (UAE) or microwave-assisted extraction (MAE), gave the best yield (4.13 ± 1.37 mg CGE/g dw) in the total anthocyanin content (TAC) from dried tepals, when compared to different MeOH/water mixtures (MeOH 20%, 50%, 80%). Contrasting results were obtained by Caser et al. [50] on the same matrix, since 80% methanol turned out to be a better solvent compared to water. However, these authors used fresh tepals instead of dried ones, and this suggests that the choice of solvents and the efficiency of extraction are also influenced by the water content of the plant matrix. Another crucial factor is the origin of the plant material, as the soil type and the climate conditions, as well as other environmental factors, lead to different phytochemical profiles of the same plant species. An outstanding example of this is the study conducted by Gigliobianco et al. [19], who compared different green solvents for the extraction of bioactive compounds from dried saffron by-products collected in two different Italian regions using MAE as a technique. For tepals from the Piemonte region, the highest anthocyanin yield was obtained with pure water, whereas for those from the Marche region, the best recovery was reached with EtOH 70%.

Pappas et al. [22] carried out a comparative evaluation of different innovative technologies for the extraction of total anthocyanins from freeze-dried saffron tepals using aqueous solutions of citric acid (CA) and lactic acid (LA) at different concentrations. The outcomes indicated that no specific pattern was detected concerning the acid type and acid concentration, and the best performance, in terms of anthocyanin extraction and antioxidant capacity, was obtained using a stirred-tank extraction with 1% (*w*/*v*) LA solution, yielding 3.25 g cyanidin-3-*O*-glucoside equivalents (CGE)/kg in the dry weight (DW) of tepals.

In two out of four studies that focused on the extraction of anthocyanins from by-products of cranberry and bilberry juice production, water was selected as the best solvent to extract glucoside-, galactoside-, and arabinoside- derivatives of delphinidin, cyanidin, petunidin, peonidin, and malvidin, both applying supercritical fluid extraction (SFE) and UAE [34,36]. The use of water acidified with either 1% LA or CA was confirmed to give a comparable TAC from the grape pomace by ohmic heating, and a better extraction yield was found compared to pure water [46]. In other cases, the type of acid was found to have a significant role in the extraction performance, as reported in early investigations for wine pomace [51] and more recent ones for red grape pomace [52]. Water was also used in combination with β-cyclodextrin for the extraction of bioactive phenolic compounds from pomegranate (*Punica granatum* L.) peel [29]. Cyclodextrins (CDs) are cyclic oligosaccharides belonging to the group of Generally Recognized as Safe (GRAS) compounds, largely used by the food industry for increasing the solubility, stability, and bioavailability of bioactive compounds [53]. Their hydrophobic cavity allows for the incorporation of several types of molecules, while their outermost, hydrophilic part enables solubilization in polar solvents, including water. The efficacy of applying the CDs and UAE technique, compared to the aqueous extraction method, was evaluated for the recovery of different classes of phenolic compounds from pomegranate peel by Kalantari and co-workers [29], using the response surface methodology (RSM) to optimize the extraction conditions. The results showed that the addition of 1.8% β-cyclodextrin to water was able to improve the extraction efficiency in terms of total phenolics, total flavonoids, total flavonols, and TAC.

### 2.2. Bio-Derived Solvents

Bio-derived solvents have recently attracted the interest of researchers because of their advantages over conventional volatile organic compounds (VOCs). They are produced from a wide array of renewable sources, including plant and aquatic biomasses, such as corn, wood, algae, or compatible waste materials from the food industry, through transformation processes occurring in biorefineries, such as fermentation or chemical transformation. Bio-solvents are characterized by high biodegradability, low toxicity [54], and rather low viscosity, which make them particularly suitable for the extraction of some classes of bioactive compounds. Moreover, bio-derived solvents have a different affinity for water; thus, they can be classified either as hydrophilic (e.g., glycerol and ethyl-lactate) or as hydrophobic (e.g., α-pinene, p-cymene or D-limonene) [55]. On the other hand, their limitations and drawbacks include cost, a high boiling point, and a generation of off-flavors [49].

As concerns anthocyanin extraction from food by-products, the bio-derived solvents that are mainly used include ethanol and glycerol.

### 2.3. Ethanol

Ethanol (EtOH) is considered to be a safe and environment-friendly solvent belonging to the GRAS substances [56]. Ethanol is easily available in high purity at reasonable prices and is completely biodegradable. It can be produced by the fermentation of plant materials rich in sugars or in polymers, such as starch and cellulose, and is called, in this case, green ethanol or bioethanol. Plant sucrose sources, such as sugarcane, sugar beet, cassava, and starchy materials, such as corn and wheat, represent the most used feedstocks, which give a high ethanol yield and productivity per area [57,58], while bioethanol production from lignocellulosic sources is more costly and less yield-productive [59].

Although ethanol is one of the solvents with the least impact on the environment, its volatility increases air emissions, and, being flammable, waste management after extraction is not easy [60]. Thus, to further reduce its environmental impact, it is advisable to mix it with water, as emerged from a comprehensive assessment of green solvents and mixtures carried out by Capello et al. [14]. These authors combined the evaluation of substance-specific hazards by using the environmental, health, and safety (EHS) method with the whole life-cycle assessment of solvents. The solvent mixtures with a high water content showed very low environmental impacts due to the given zero score of EHS and the low cumulative energy demand (CED) for this solvent. Thus, the higher the water percentage in the mixture, the lower its environmental impact.

In our article collection, several investigations used a mixture of ethanol/water as green solvents to extract anthocyanins from different by-products [18,24,25,26,32,33,35,40,41,42,61], and the combination of the two solvents turned out, in most cases, more efficient compared to the individual ones. The percentage of ethanol used in the mixtures ranged from 20% to 70%. The lowest percentage (20%) was employed for the recovery of anthocyanins from the red grape pomace by SFE, even though a better yield (up to ten-fold higher) was obtained on the same matrix with EtOH 50% using pressurized liquid extraction (PLE). Hence, in terms of effectiveness, the lowest ethanol percentage used in our article collection was 30%, which was applied to treat pomegranate by-products (peel and male flowers) by UAE [28] and black bean (*Phaseolus vulgaris* L.) by-products employing both PLE and UAE [25]. However, most reports used 50% ethanol [26,33,35,40]. In three studies [24,26,32], the percentage of ethanol giving the best yield was established by constructing a statistical model through the response surface methodology (RSM) approach, and a percentage between 47.5 and 55.6 gave the best performance, even with different matrices and using different techniques. A mixture of ethanol/water 60:40 (*v*/*v*) was used by Posadino et al. [42] to extract anthocyanins from the pomace of *Vitis vinifera* L. var. *Cagnulari* using the technology of a Naviglio^®^ extractor, and a yield comparable to that with SFE was obtained, even though it was expressed in terms of the total polyphenol.

A higher proportion of ethanol in the solvent mixtures had a negative impact on the antioxidant extraction. This is general evidence, as several authors reported that when the percentage of ethanol in the mixture was increased at fixed temperature and extraction times, the yield of the anthocyanins gradually increased up to a certain point and, thereafter, decreased [24,32]. This could be due to two reasons: on one side, high percentages of ethanol decrease the polarity of the extraction mixture, reducing the solubility of anthocyanins, and on the other side, high concentrations of ethanol cause the denaturation and the precipitation of proteins into the cells, thereby preventing the extractability of phenolic compounds from the plant matrix. Obviously, the best optimal percentage of ethanol to be used greatly depends on several variables, such as the extraction technique, the extraction time, the liquid–solid ratio, the pH of the mixture, and the type of plant material, among others. However, it is advisable to select a mixture with a low ethanol/water ratio to meet the requirements of a green approach in order to have a low environmental impact. The highest percentage of ethanol (70%) was tested by Li et al. (2014), which found it more effective for recovering anthocyanins from saskatoon (*Amelanchier alnifolia* Nutt. ex M.Roem.) pomace compared to pure water using the UAE technique. The same results were obtained by Gigliobianco et al. [19] when applied to MAE, but only for one of the two saffron varieties tested, as mentioned above.

### 2.4. Glycerol

Glycerol is a simple alcohol that is widespread in nature, as it forms part of the chemical structure of fats and oils. It can be produced either by chemical synthesis from petrochemical feedstocks, as a by-product of the soap manufacture and bio-diesel industries, or by microbial fermentation. Being non-toxic, non-flammable, non-volatile under normal atmospheric pressures, and biodegradable [62], it is used as a food additive, sweetener, preservative, and in cosmetic and pharmaceutical formulations. Moreover, it is available on a large scale from the vegetable oil industry.

The main limitations to the use of glycerol relate to its viscosity at room temperature, which may affect the mass transfer from the plant matrix. In addition, the hydroxyl groups are reactive and can lead to the formation of side products if the extraction takes place under acidic or basic conditions [63]. Furthermore, glycerol has a low vapor pressure that prevents its removal through evaporation. This could be solved by using glycerol both as a solvent and as part of the final formulation, which would also reduce the number of steps to obtain the final product, thus saving energy.

In our article collection, different percentages of glycerol were employed for anthocyanin recovery. Gigliobianco et al. carried out extraction from saffron tepals collected in two Italian regions using 100% glycerol and found that the efficiency varied, compared to an ethanol/water mixture, depending on the sample origin [19]. In a study carried out by Eyiz et al. [43], the glycerol concentration and solid–liquid ratio were evaluated as independent factors for the optimization of the recovery of several classes of phenolic compounds from grape (*Vitis vinifera* L.) pomaces, including the total monomeric anthocyanins. Glycerol at 50% and a solid–liquid ratio of 22.4 maximized the extraction of all the targeted secondary metabolites. Katsampa et al. [17] applied a Box–Behnken experimental design for optimizing the extraction of colored pigments from red onion cataphylls for the valorization of *Allium cepa* L. by-products. The results showed that increasing the glycerol concentration up to 50% produced a higher anthocyanin content in the extract. This was probably due to the lowering of the dipole moment of the extraction mixture, which has a positive effect on the recovery of these types of molecules. These authors found that the optimal glycerol concentration was 83% (*w*/*v*), using UAE at 80 °C and with a solvent-to-mass ratio of 88:1. Mourtzinos et al. [64] improved the extraction of anthocyanins and flavonols from onion solid wastes by adding 2-hydroxypropyl-β-cyclodextrin (hp-β-CD) to aqueous glycerol solution. Precisely, the optimal conditions were glycerol 60% (*w*/*v*) and hp-β-CD 6.5% (*w*/*v*). The extractive yield was 75% higher than that obtained with an ethanol extraction and the onion leaf extract proved to be a stable natural colorant for yogurt matrix due to the presence of anthocyanins.

### 2.5. NaDES

Natural deep eutectic solvents (NaDES) have been increasing in popularity over the last two decades as promising candidates for the green extraction of bioactive compounds from several plant materials since, by varying polarity, viscosity, and extraction temperature, they can be successfully used for the recovery of both polar and non-polar natural products [65,66,67,68,69,70]. They represent a new class of ionic liquid analogs, generally based on mixtures of natural compounds, usually obtained by the complexation of quaternary ammonium salt (working as Hydrogen Bond Acceptor, HBA) with a metal salt or hydrogen bond donor (HBD); this complexation causes the delocalization of the electric charges, resulting in a decrease in the melting point in the mixture relative to the melting points of the individual components [71,72]. The preparation of the eutectic mixture can be carried out through several methods, namely heating and stirring [73], evaporation [74], freeze-drying [47,75], microwave [76,77], or ultrasound-irradiation [76,78]. In terms of energy consumption, the preparation of NaDES using microwaves and ultrasounds can be considered more environment-friendly than the other methods since both techniques require significantly less time for the formation of the mixture. The stability of NaDES depends on the number of hydrogen bonds and their spatial structure, which also determine the extraction capacity towards the target compounds.

Most studies use choline chloride (ChCl) as a quaternary ammonium cation, while different HBDs have been evaluated to date, including amino acids, sugars, carboxylic acids, and alcohols. Since these components are primary metabolic substances naturally present in all living organisms, and the formation of intracellular eutectic mixtures has been demonstrated during specific plant developmental stages [79], NaDES are generally considered to be green solvents with high biodegradability and low toxicity, thus having great potential in pharmaceutical, cosmetic, and food-related applications [80,81]. However, limitations to the use of these solvents can derive from the still scarce information regarding their toxicity and environmental impact. Since NaDES have special physicochemical properties compared to the individual components, it is important to carefully evaluate their toxicity and cytotoxicity before truly claiming them as nontoxic and biodegradable solvents. As demonstrated by Hayyan et al. [82,83], the toxicity and cytotoxicity of the mixture were higher compared to that of individual components, although it greatly varied depending on their structure, and the results pointed to the role of HBDs (organic acids) as a major enhancer of cytotoxicity. The state-of-the-art microbial toxicity of NaDES towards both prokaryotic and eukaryotic organisms has been recently reviewed by Marchel et al. [84]. Besides the paucity of studies on this topic, contrasting reports exist on the biocompatibility of these mixtures due to the lack of adequate and standardized methodologies for NaDE’s toxicity determination. Thus, more investigation is needed on this aspect. In view of that, the evaluation of the cytotoxicity of NaDES, when used for bioactive extraction, should always be conducted from the perspective of their use for pharmaceutical, nutraceutical, and cosmetic applications. A similar approach was followed by Radosevic et al. [85], who made a preliminary evaluation of NaDES’s cytotoxicity on human cell lines prior to their use for preparing an anthocyanin-rich extract from the grape skin. In addition, problems could arise due to the viscosity of NaDES, easily be solved by mixing them with water.

Different types of NaDES have been tested for the extraction of anthocyanins from agri-food by-products. ChCl was the most widely used ammonium cation in our selected article collection, while organic acids were mainly employed as HBDs, due to their combined polarity and acidity, which favor the extraction and the stability of anthocyanins. In the article collection here examined, NaDES were used for the recovery of anthocyanins from by-products derived from red grapes [39,44,47], blueberries [37], and jabuticaba fruit [86]. In all studies, it emerged that the molar ratio among the components of the eutectic mixture, the amount of added water, and the solid-to-liquid ratio had a marked impact on the extractive capacity of NaDES. In general, an increase in the water content in the mixture caused a decrease in its viscosity, which improved the mass transfer rate between the solid and the liquid phase and thus facilitated the extractability of anthocyanins from the matrix. However, excessive water incorporation interferes with the HBD-HBA system, reducing the interaction between the NaDES and anthocyanins, thus decreasing the extraction yields. In general, the percentage of water added to the eutectic mixture varied in the different studies, depending on the applied techniques and matrices, but most research used about 30% water [37,39,45,47]. The water content was higher when PLE was applied due to the characteristics of this technique, for which solvents with a low viscosity are required [44,86]. For the recovery of anthocyanins from the red grape pomace, the eutectic mixture composed of ChCl and an organic acid turned out to be the most effective compared to the other HBD-HBA systems screened [44,45]. Specifically, the best extraction yield from the grape pomace of Croatian native *Vitis vinifera* cv. Plavac mali was reached with ChCl:CA (2:1) and ChCl:Proline:Malic acid (1:1:1) with 30% (*v*/*v*) water using an ultrasound microwaves assisted extraction (UMAE) [45]. Nevertheless, the evaluation of the stability of the extracts led to the selection of the ChCl:CA mixture since that extract showed better preservation at 4°C and −18 °C. In addition, the lower cost of the mixture was an added value for scaling up the procedure to an industrial level, and this aspect should also be considered when proposing a method for such purposes.

In a screening of NaDES for recovering anthocyanins from the grape pomace of *Vitis vinifera* L. cv. ‘Tempranillo’, Loarce et al. [44] found that the mixture ChCl:OA (1:1) gave the best results using PLE, while the mixture ChCl:MA (1:1) with 35.4% water was selected as the best performing by Bosiljikov et al. [89] using UAE for the extraction from the lees of the red grape. In another study on red grape skin using UAE, CA:Maltose (4:1) and 23.8% water gave a higher yield compared to both the other tested eutectic mixtures and the most common reference solvents [47]. These findings highlight how the extraction efficiency can be different in the various by-products obtained from the same plant material, which is probably due to their different anthocyanin compositions, and underlines that the best performance is obtained by the combined action of the solvent mixture and the applied technique. The NaDES mixture ChCl:OA (1:1) proved to be the best choice for the blueberry pomace using UAE [37], while ChCl:MA (1:1) again emerged as the most suitable for the recovery of anthocyanins from jabuticaba peel by PLE, giving a 50% higher extractive yield compared to water and a more stable extract with respect to that obtained with ChCl:Pro (1:2) [27].

## 3. Green Techniques for Anthocyanin Extraction from Agri-Food By-Products

For an extraction process to be truly claimed as “green”, it is important to combine the use of green solvents with the application of innovative techniques, which allow the reduction in the consumption of solvents, time, and energy, while preserving the stability of the obtained product. This is particularly important for anthocyanins, which are thermo-sensitive molecules.

Emerging from the bibliographic survey, the most used green techniques for anthocyanin recovery from agri-food by-products were the ultrasound-assisted-extraction, microwave-assisted extraction, and pressurized liquid extraction. Less used, but still effective were supercritical fluid extraction (SFE), electric treatments (Pulsed Electric Field and High Voltage Electrical Discharge) enzyme-assisted extraction (EAE), homogenizer-assisted extraction (HAE), and ohmic heating-assisted extraction (OHAE).

### 3.1. Ultrasound-Assisted Extraction

UAE is considered a green technique for its short extraction time and low energy input. In addition, UAE allows for the use of small volumes of green solvents since it improves the extraction performance.

It is based on a complex mechanism in which ultrasounds cause the acoustic cavitation effect that triggers several physical and mechanical events acting on the plant matrix. In particular, bubbles generated by cavitation collapse and release high energy, thus generating macro-turbulence, micro-mixing, shear forces, shock waves, and microjets. These events act on the sample by increasing the surface area thanks to fragmentation, erosion, peeling, and particle breakdown. Thus, the penetration of the solvent into the matrix is fostered, as well as the matrix hydration and swelling, and the mass transfer of target metabolites into the solvent is favored, thanks to the generated increase in the temperature in the liquid system [87]. Under these conditions, highly reactive radicals can be formed, which may degrade the compounds of interest [88], and thus, care should be taken with the ultrasound intensity and the duration of treatment used.

UAE represents one of the best technologies from an environmental point of view and allows an improved extraction process with high energy efficiency and low energy costs. The environmental impact of UAE, in comparison with that of maceration and Soxhlet extraction, was evaluated by Chemat et al. [88] in terms of both energy consumption and CO_2_ released in the atmosphere. The electrical energy required for the ultrasound supply was 0.25 kW/h for UAE, 6 kW/h for Soxhlet, and 8 kW/h for maceration, and the amount of CO_2_ poured into the atmosphere was much lower for UAE compared to the other two conventional techniques (18 and 32 times lower than maceration and Soxhlet, respectively). Therefore, UAE has recently become attractive for several industries to obtain bioactive compounds from different plant matrices [89].

In our collected articles, UAE emerged as the most used technique for the extraction of anthocyanins from agri-food by-products. The extraction time ranged from between 3 and 90 min. The shortest extraction time (3 min) was applied for the recovery of the bioactive compounds (anthocyanins, polyphenols, tannins) from jabuticaba peels using high-intensity ultrasound-assisted extraction (HIUS-AE), with high-intensity ultrasounds of 3.7 W/cm^2^ and a water/ethanol mixture as a solvent [26]. Short extraction times are mandatory for HIUS-AE to avoid the possible degradation of anthocyanins. Varo et al. [36] found that the maximum anthocyanin extractive yield from bilberry juice by-products was reached after 5–7 min of HIUS-AE at 16.7 W/cm^2^, while the yield was reduced when extending the extraction for up to 60 min. This is probably due to the formation of free radicals, which increase the polymerization and depolymerization reactions, and to the increase in temperature caused by the process intensification, which strongly affected the stability of the anthocyanins [11]. Another application of UAE with very short times is the pulsed mode (P-UAE) [30,37]. Fu et al. [37] obtained the best anthocyanin extraction yield from blueberry pomace in 3.2 min with 325 W of ultrasonic power, 76 °C, and 60:1 NaDES as a solvent-to-mass ratio. Extraction time can be extended when using lower ultrasonic power. The longest extraction time of 90 min was reached with an ultrasonic power of 64 W [33]. Regarding the frequency, low values are preferred since they favor a better formation of cavitation bubbles, keeping the amount of the formed radicals at a low level [90]. In our selected articles, the frequency ranged between 23 and 40 kHz, although not all of them reported this piece of information in the paper. Nevertheless, it is important to consider that ultrasonic power and frequency are not the only determinant factors for the extraction process since other parameters, such as the solvent-to-solid ratio, the temperature, and the type of matrix, also play an important role. The applied solvent-to-mass ratio was variable, ranging between 4:1 and 200:1 (*v*/*w*). However, several studies for the optimization of the UAE method reported better results with a higher solvent-to-solid ratio since the mass transfer from matrix to solvent is promoted under these conditions [17,33,39]. When using UAE as a technique, the temperature ranged between 21 °C and 80 °C. The general trend was that raising temperatures increased the anthocyanin yield up to a certain point, after which the degradation processes took place [24,29]. Therefore, this parameter must be carefully monitored.

### 3.2. Pressurized Liquid Extraction

PLE, also known as pressurized solvent extraction, is a technique based on the use of solvents at high temperatures and pressures. It is considered a green technique because it allows a small consumption of safe solvents, such as water, and a short extraction time. Thanks to the high pressures used, the extraction can be carried out at a high temperature without solvent evaporation. Under these conditions, the solvent viscosity is decreased, the solvent penetration into the plant matrix is promoted, and the mass transfer rate and the solubility of the metabolites are enhanced, thus improving the extraction performance [91]. Thus, when using PLE, it is essential to select the appropriate solvent and temperature to obtain the right solvent polarity and extract the desired metabolites.

From our bibliographic survey, five studies exploited the PLE technique with green solvents to extract anthocyanins from different matrices, such as saffron [22], black bean [25], jabuticaba [27], and red grape [40,41,44]. All studies agreed that the pressure did not significantly affect the extraction yield, while the temperature was the most important variable since it influenced both the polarity and the solubility. Thus, care should be taken to avoid the thermal degradation of anthocyanins. Teixeira et al. [25] and Loarce et al. [44] tested different temperatures using ethanol:CA solution and NADES as the solvent mixtures, respectively, and they both obtained the best recovery at 60 °C. Using this temperature, Teixeira et al. [25] obtained a higher yield compared to other applied techniques, such as UAE and MAE.

Other authors choose a higher temperature (120 °C) to extract anthocyanins [22,40,41]. This may contradict the above-mentioned results, but extraction parameters, such as time and the number of extraction cycles, must be considered. It has to be noted that Pappas et al. [22] and Poveda et al. [41], extracting at this high temperature, obtained a lower yield compared to the other techniques they used, such as UAE, heating, or stirring. Anyway, in these works, the extraction method has been optimized for the total phenolic content and not for anthocyanins only; thus, it is not possible to assess whether the best-selected extraction conditions would also prove optimal for anthocyanins.

Regarding the extraction time, all studies performed the extraction with short extraction times (10–26 min) [22,25,41,44], except Otero Preja et al. [40], who carried out the extraction for 90 min. The possibility of using short extraction times offered by the application of this extraction technique represents an undoubted advantage in terms of energy consumption and allows its claim as a green method.

### 3.3. Microwave Assisted Extraction

MAE is an environment- and user-friendly technique since it allows low solvent consumption and short extraction times, thus generating low masses of waste. Thanks to these features, users are less exposed to solvents, and the release of harmful chemicals into the environment is minimized. In MAE, samples and solvents are heated by the application of two orthogonal oscillating fields, magnetic and electric. In polar solvents, a friction force is generated by both the compelled movements of the dipoles interacting with other polar components in order to align with the applied electromagnetic field and the consequent movement of the molecules through the solution, which also contributes to generating resistance. This friction force produces heat. Conversely, non-polar solvents remain transparent to microwaves and thus do not generate heat [92]. In the plant matrix, this friction force is generated by the plant moisture, which heats up and evaporates; consequently, the pressure inside the cell increases, the cell wall collapses, and the cellular sap with metabolites spills out. However, special attention should be paid to the uniformity of the heating process, which is not easy to achieve and for which modeling studies are still lacking [93].

The efficiency of MAE depends on several factors: solvent composition, extraction time, microwave power, temperature, and intrinsic properties of the plant material. In our search, four studies selected MAE as a green extraction technique. Doulabi et al. [32] optimized it for the extraction of anthocyanins from eggplant peels. They found that when increasing the microwave power from 100 to 300 W, the total anthocyanin content was higher since microwave power improves the solvent penetration into the plant matrix. However, the parallel increase in temperature, generated by the microwave energy, was able to increase the extraction only until a certain point, after which degradation processes were triggered. This represents a critical aspect of this technique since excessive heating could compromise the recovery of thermolabile analytes. For this reason, extraction time is an important parameter to control in order to avoid overheating. The same authors obtained better extractive yields by lowering the solvent-to-solid ratio, probably because a higher amount of solvent reduces the microwave energy absorption by the solid since the solvent itself absorbs most of the microwave energy. In accordance with these observations, the response surface methodology (RSM) for the optimization of the extraction conditions gave parameters of 298.84 W for the microwave power, 5.78 min for the extraction time, 6.4:1 for the solvent-to-solid ratio, 55.56% (*v*/*v*) of ethanol as the solvent and a pH of 4.57 [32]. A similar solvent-to-solid ratio (5.5:1) was applied by Gigliobianco et al. [19] for the recovery of bioactive compounds from saffron tepals, using a magnetron operating at 2.45 GHz for 30 min at 70 °C [19]. Panić et al. [45] compared the efficiency of UAE, MAE, and their combination, namely, ultrasound/microwave-assisted extraction (UMAE) in recovering anthocyanins from red grape pomaces for industrial applications. The small-scale experiments showed a higher efficiency of UMAE compared to MAE and UAE. The irradiation with ultrasounds and microwaves (MW) in sequence led to a remarkable increase in the extraction yield, suggesting a positive synergistic effect of the combined treatment. Probably, ultrasounds caused the breakage of the plant cell structure, and the MW treatment favored the release of the active compounds into the solvent. Moreover, the combination of ultrasounds with microwaves can reduce the excessive heating caused by microwaves in certain areas of the treated sample. The authors optimized the method for large-scale experiments by RSM, demonstrating that a UMAE extraction of 15 g of grape pomace in 500 mL of NADES with 30% (*v*/*v*) water, using US pre-treatment for 5 min at 500 W, followed by 10 min of MW irradiation at 300 W, gave an extraction yield, calculated as the sum of the target anthocyanins detected by HPLC, almost equal to the predicted one (above 1.8 mg/g dw).

### 3.4. Supercritical Fluid Extraction

Supercritical fluid extraction (SFE) is based on the use of supercritical fluids, substances for which both the temperature and pressure are above the critical point. Under these conditions, the interface between the liquid and vapor state decreases, and the substance is neither gaseous nor liquid. A supercritical fluid (SF) possesses a gas-like viscosity and fluid-like density, and its compressibility changes drastically even with a small variation in temperature and pressure. Therefore, the solubility can be easily changed during the extraction process [94]. The use of supercritical fluids avoids the use of organic solvents, but high pressure is required, resulting in high operating costs due to increased safety requirements. The most commonly used substance for SFE is carbon dioxide since it is non-flammable, inert, has low toxicity, and can be produced as a by-product of biogas production or the fermentation processes [48]. However, having CO_2_ an intermediate polarity between non-polar and low-polar solvents, it is poorly suitable for the extraction of polar compounds such as anthocyanins. One way to overcome this issue is to use co-solvents that increase the polarity of the extractive mixture, as conducted in the research of our article collection.

Otero Preja et al. [40] used CO_2_ and 20% ethanol (*w*/*w*) as a co-solvent for the extraction of anthocyanins from the pomace of different varieties of red grape, and they compared SFE with PLE using ethanol. The extractive performance, in terms of both global yield and the yield of anthocyanin and phenolic compounds, was lower with SFE compared to PLE, the difference being five- to thirty-fold [40]. Kühn and Temelli [34] compared the efficiency of SFE using CO_2_ + water with that of the ternary mixture CO_2_ + ethanol + water for recovering anthocyanins from cranberry pomace, and they reported that just water as a co-solvent gave the best anthocyanin yield. This is probably because water causes a decrease in pH due to the in-situ formation of carbonic acid, which might increase the cell membrane permeability and the stability of the target compounds [34]. Hence, achieving a sufficient polarity of the extracting mixture is the most critical aspect, which makes SFE less appropriate for anthocyanin extraction than the other green techniques.

### 3.5. Electric Treatments

Electric treatments, such as high voltage electrical discharge (HVED) and pulsed electric field (PEF), are non-thermal processes based on different extraction principles, which preserve the quality of the extracted components. HVED is a technique that allows a good performance to be obtained using low energy input and low organic solvent volumes. In this technique, the extraction takes place through two sequential distinct steps: a pre-breakdown phase, followed by a breakdown one. In the former, a stream of ionized vapor channels propagates from the tip points inserted into the extraction chamber toward the opposite electrode, and the second phase begins as soon as the stream reaches the electrode. In this phase, an electrical arc passes through the previous stream. In both phases, gaseous bubbles are produced, and cavitation occurs. In addition, during the breakdown phase, a shock wave and chemically reactive species are produced. Because of this shock wave, cavitation occurs, and turbulences are observed, thus producing the fragmentation of the raw material and promoting extraction [95]. For these reasons, the breakdown phase is the most important for polyphenol extraction [96,97]. However, high input energy could compromise the final extraction yield since the radical species produced in these conditions can damage the metabolites of interest. Hence, the energy needs to be carefully selected to only improve the cell disruption process without oxidizing the bioactive compounds [35,97]. A limitation of this technique is its low selectivity, as it destroys both cell membranes and cell walls, thus resulting in the release of several metabolites. In contrast, PEF, which only acts on membranes, is more selective [98].

PEF technology is based on cell permeabilization, which promotes mass transfer by making the metabolites spill over. It is a low-energy consumption process where a short pulsed electric field generates a temporary destabilization of the cell membrane due to its charging and polarization. This disturbance in the cell membrane structure forms pores through which metabolites pass. If the electric field is strong, the electroporation becomes irreversible since the cell membrane breaks down. In this technique, several parameters, i.e., the strength of the electric field, the number of pulses, their duration, and frequency, must be considered because they influence the electroporation process and, therefore, the extraction yield [99].

In our survey, only one study applied HVED to extract anthocyanins from the blueberry pomace [35]. The extraction was performed using EtOH 50% as a solvent at 30 kV, 20 °C, and 100 Hz for 15′, with a liquid-to-solid ratio of 50:1 and a 5 mm distance between the electrodes. Under these conditions, a yield of 1087.18 µg anthocyanins/g dw was reached (Table 1), which turned only slightly lower than that obtained with MeOH 50% (1221.28 µg/g), thus highlighting that this extraction method can be successfully applied using green solvents.

The same authors also performed a PEF-assisted extraction using EtOH 50% with 1% of HCl as a solvent and a liquid-to-solid ratio of 50:1; hence, the extraction was carried out with 100 pulses under an electric field intensity of 20 kV/cm. Under these conditions, the yield was 1624.54 µg anthocyanins/g dw, slightly lower than that obtained with MeOH 50% with 1% of HCl as a solvent (1757.32 µg/g). Just as the HVED-assisted extraction, this yield difference highlights that this technique combined with green solvents can be successfully used to recover anthocyanins from blueberry pomace, and comparing the two electric treatments, PEF turned out to be more efficient than HVED, and both were, in turn, more efficient than UAE. In addition, it should be noted that the application of these two extraction methods could be further improved, taking into consideration that the authors focused on the global extractive yield and not specifically on the anthocyanin yield. Although promising, these techniques do not have the same energy impact when applied at an industrial scale, as the energy required to obtain yields comparable to the lab scale is much higher for processing large masses of by-products [100].

### 3.6. Other Techniques

EAE is a green technique based on the use of hydrolytic enzymes, which contribute to the improvement of metabolite release from the plant material, therefore improving the final yield. Hydrolytic enzymes, such as cellulases, pectinases, and hemicellulases, degrade the polysaccharidic polymers of the cell wall, thus loosening and disrupting the network structure. Target metabolites are released from both the inner cell environment and the cell wall since they are also retained in the wall matrix linked through hydrogen or hydrophobic bounds. To optimize EAE, several parameters, such as the time of incubation, temperature, pH, enzyme type, and enzyme concentration, should be considered. In our survey, Vardakas et al. [21] used pectinolytic, cellulolytic, and hemicellulolytic enzymes for recovering anthocyanins from the saffron tepals. The 1:1 binary enzyme combination of cellulase and hemicellulase preparations gave the best results, improving the anthocyanins extraction yield by 38% with respect to the sample without enzyme treatment. The enzyme dose and incubation time markedly influenced the effectiveness of the extraction since the anthocyanin yield was raised to a certain point by increasing the enzyme percentage, thereafter declining. This is probably due to secondary enzyme activities that led to the hydrolysis of the anthocyanidin glycosides, converting them into more unstable aglycones. The optimization of these independent variables was reached through RSM, which allowed the selection of the best conditions at a 0.12–0.15% enzyme dose/100 g substrate and 145–185 min extraction time. Despite the proven effectiveness, EAE may not be considered a technique of choice for the treatment of huge amounts of agri-food by-products since enzyme kinetics may vary depending on the percentage of dissolved oxygen, temperature, and nutrient availability, which can be different from a small-scale to large-scale. Moreover, in the latter case, the high cost of enzyme mixtures should also be considered.

HAE provides for the use of a homogenizer, performing high-speed cuts on the plant material, which provokes mechanical damage that facilitates the release of the metabolites. The solid–liquid ratio, determining the solvent-sample interface, and the polarity of the extractant mixture have a strong impact on the extraction. Compared to traditional extraction methods, HAE is a green technique since the high shear rate speeds up the release of the target compounds, reduces the use of solvents, and leads to a considerable shortening in the extraction time. In addition, shearing replaces system heating, which is used to improve the yield of conventional extractions, resulting in further energy savings [101]. In our article selection, HAE was applied by Eyiz et al. [43] for the recovery of anthocyanins from red grape pomaces, using glycerol as a solvent. These authors carried out a double homogenization, first at 10,000 rpm for 30 s and then at 15,000 rpm for 30 s. By means of RSM, the extraction conditions were optimized by setting the percentage of glycerol in water at 50% and the liquid-to-solid ratio at 22.4. The simplicity of this technique makes it easily applicable for processing large quantities of by-products.

OHAE involves a passage of electricity through the sample, which heats it up internally. Since plant matrices consist of water, salts, and organic acids, which make them into semiconductor materials, this technique proves rather effective. The electric field can cause changes in the permeability of plant cell membranes; hence the diffusion of metabolites is increased, and the extraction of bioactive compounds is improved [102]. Compared to the externally induced heating of traditional extractive methods, the heating of OHAE is much more rapid, uniform, and less aggressive, thus preserving thermolabile molecules such as anthocyanins and consuming less energy. Coelho et al. [46] compared OHAE with conventional methodologies for anthocyanin extraction from red grape pomaces. Specifically, grape by-products were pre-treated with 0.1 M NaCl solution to increase the conductivity, then one part was subjected to OHAE, another one was heated traditionally, and a third part was left at room temperature. By applying an electric field of 30 V/cm and a high frequency of 25 kHz, the temperature reached 100 °C in 13 s, while with conventional heating, 20 min was required. In the resulting samples, anthocyanins were extracted with water, acidified water, and an acidified methanol solution. The authors denoted a greater anthocyanin recovery with OHAE than with conventional methods; hence, OHAE was able to reach a better extractive yield, saving time and energy.

## 4. Concluding Remarks and Future Perspectives

This review highlights that the application of green techniques has become an increasingly frequent approach in the last decade for recovering anthocyanins from agro-industrial by-products. The use of innovative procedures, such as UAE and MAE, their combination, and PLE allows, in most cases, anthocyanin-rich extracts to be obtained with great efficiency in a very short time, with the low consumption of solvents, and with a low environmental impact. Although general guidelines can be drawn, the studies collected highlight that several factors should be considered when applying a green procedure for the recovery of this class of antioxidants. First, consideration should be given to the plant matrix from which the extraction is to be carried out, i.e., the type of anthocyanins that are expected to recover, so as to obtain information about their polarity, bearing in mind that the same plant species from which by-products are derived may have a different phytochemical profile due to different growth or harvest conditions. Another critical factor is the water content of the source material; working on fresh or dry material determines the greater success of some methodologies rather than others. Moreover, given the sensitivity of anthocyanins to temperature, an efficient extraction should avoid high temperatures.

As a general conclusion, the effectiveness of anthocyanin extraction is the result of a combination between the solvent used and the applied technique. However, it is worthwhile to underline that the efficiency yield does not represent the only aim when selecting the best green technology to be used since other important aspects, such as energy cost, safety, and environmental impact, should also be taken into consideration.

The analysis of the collected literature highlights that UAE represents one of the most suitable techniques for this class of flavonoids, which offers several advantages in terms of yield, selectivity, extraction time, and safety. However, it also demonstrates that most research used this technique on a lab scale, while its translation to a large-scale recovery is still limited. Thus, it is of primary importance, in the near future, to broaden the application of this and other innovative techniques for commercial uses, besides exploiting other plant matrices, which are, at present, poorly used, such as red apples, plums, cherries, blackcurrants, red currants, elderberries, and strawberries.

## Figures and Tables

**Table 1:** Summary of the results emerging from the survey showing the green extraction procedures applied for recovering anthocyanins and the obtained yields. The table reports: the plant source, the by-products, the green solvents used, the green technique applied, the extraction conditions, the type of anthocyanins analyzed, the extraction yields and the corresponding reference.

Plant Species	Common Name	By-Product	By-Product Origin	Green Solvent	Green Technique	Extraction Conditions	Type of Anthocyanin	Extraction Yield	Ref.
*Allium cepa* L.	Red onion	Solid wastes	Industrial processing	Glycerol	UAE	Solid–liquid ratio 1:88 (g/mL), glycerol 83% (*w*/*v*), 80 °C, 60 min, 140 W, 35 W/L, 37 kHz	Cyanidin-derivatives	2.09 CGE mg/g dw	[17]
*Amelanchier alnifolia* Nutt. ex M.Roem.	Saskatoon	Pomace	Juice production	Ethanol	UAE	5 g of sample, 25 mL of EtOH 70%, 10 min, twice. Final extraction with 25 mL of 0.15 N HCl	Cyanidin-, Delphinidin-derivatives	TMA = 2.6 ± 0.1 mg CGE/g dw	[18]
*Crocus sativus* L.	Saffron	Tepals	Flower processing	Ethanol	MAE	4.5 g of sample, 20 mL EtOH 70%, 70 °C, 30 min, 2.45 GHz	Delphinidin-, Malvidin-, Petunidin-derivatives	1.86 mg DGE/g dw for sample from Marche region (Italy) 0.35 mg DGE/g dw for sample from Piemonte region (Italy)	[19]
				Glycerol	MAE	4.5 g of sample, 20 mL Glycerol, 70 °C, 30 min, 2.45 GHz	Delphinidin-, Malvidin-, Petunidin-derivatives	0.86 mg DGE/g dw for sample from Marche region (Italy) 1.00 mg DGE/g dw for sample from Piemonte region (Italy)	[19]
				Solvent free	Cold pressing	Press	Delphinidin-, Petunidin-derivatives	1075.9 ± 20.2 mg/L from 24 h post-harvesting tepals. 1316.7 ± 109.8 mg/L from 48 h post-harvesting tepals	[20]
				Water	EAE	Solid–liquid ratio 10:1 (*v*/*w*), HCl (pH = 4), acidified binary combination of cellulolase/hemicellulase (1:1), enzyme mixture dose (0.12–0.15%), 50 °C, 145–185 min	n.s.	TMA = 2 mg CGE/g dw	[21]
					MAE	4.5 g of sample, 20 mL water, 70 °C, 30 min, 2.45 GHz	Delphinidin-, Malvidin-, Petunidin-derivatives	1.33 mg DGE/g dw for sample from Marche region (Italy) 1.18 mg DGE/g dw for sample from Piemonte region (Italy)	[19]
					PLE	Solid–liquid ratio 1:40 (g/mL), LA 5% (*w*/*v*), 120 °C, 10 min	Delphinidin-, Petunidin-derivatives	2.00 mg/g dw	[22]
					UAE	Solid–liquid ratio 1:50 g/mL, water, 21 °C, 15 min, 23 kHZ	n.s.	4.13 ± 1.37 mg GGE/g dw	[23]
					UAE + SE	UAE = Solid–liquid ratio 1:40 g/mL, LA 5% (*w*/*v*), <37 °C, 15 min, 550 W, 37 Hz, SE = 80 °C, 180 min, 500 rpm	Delphinidin-, Petunidin-derivatives	3.11 mg/g dw	[22]
*Nitraria tangutorun* Bobrov.	-	Seed meal	Seed oil factories	Ethanol	UAE	1 g of sample, 15 mL EtOH 47.49%, 70 °C, 25.3 min, 300 W, 30 kHz	Cyanidin-, Delphinidin-, Pelargonidin-derivatives	0.65 mg CGE/g dw	[24]
*Phaseolus vulgaris* L.	Black bean	Hulls	Harvesting and processing	Ethanol	PLE	5 g of sample, EtOH:CA 0.1 M = 30:70 (*v/v*), 60 °C, 26 min, flow rate 4 mL/min	Delphinidin-, Malvidin-derivatives	3.96 ± 0.20 mg CGE/g dw	[25]
					UAE	Solid–liquid ratio 1:20 g/mL, EtOH:CA0.1 M 30:70 (*v/v*), 60 °C, 26 min, 55 kHZ	Cyanidin-, Delphinidin-, Malvidin-derivatives	3.28 ± 0.22 mg CGE/g dw	[25]
*Plinia cauliflora* (Mart.) Kausel	Jabuticaba	Peel	Juice, jam and liquor productions	Ethanol	UAE	Solid–liquid ratio 1:25 g/mL, EtOH 50%, 3 min, 3.7 W/cm^2^, 19 kHz	n.s.	287.00 ± 12 mg/L	[26]
				NaDES	PLE	5 g of sample, [(ChCl:Pro = 1:2):water = 47:53] (*v*/*v*), pH 4.5, 90 °C, 5.3 mL/min	Cyanidin-derivative	TMA = 1.70 ± 0.06 mg CGE/g dw	[27]
						5 g of sample, [(ChCl:MA = 1:1):water = 47:53] (*v*/*v*), pH 1.5, 90 °C, 10 MPa, 5.3 mL/min	Cyanidin-derivative	TMA = 1.60 ± 0.09 mg CGE/g dw	[27]
				Water	PLE	pH = 6.7 or pH = 1.5, 90 °C, 10 MPa, 5.3 mL/min	Cyanidin-derivative	1.13 mg/g dw	[27]
*Punica granatum* L.	Pomegranate	Male flowers	Orchards management	Ethanol	UAE	Solid–liquid ratio 1:100 g/mL, EtOH 30%, 50 °C, 15 min, 59.2 W/cm^2^	Cyanidin-, Pelargonidin-derivatives	Different concentrations of the same variety harvested in different years	[28]
		Peel	Industrial processing	Ethanol	UAE	Solid–liquid ratio 1:100 g/mL, EtOH 30%, 50 °C, 15 min, 59.2 W/cm^2^	Cyanidin-, Pelargonidin-derivatives	Different concentrations of the same variety harvested in different years	[28]
			Fruit processing	Water	UAE	Solid–liquid ratio 1:1 g/mL, β-Cyclodextrin 1.8%, 55.7 °C, 15.38 min, 100 W, 40 kHz, dark conditions	n.s.	0.52 mg CGE/g dw	[29]
			Juice production	Water	UAE	Solid–liquid ratio 1:40 g/mL, <65 °C, 10 min, 200 W, 26 kHz, pulse duration and pulse interval ratio, 4:1	n.s.	0.6 ± 0.1 CGE/g dw (var. Akko) 0.05 ± 0.02 mg CGE/g dw (var. Wonderful)	[30]
*Rubus* spp.	Blackberry	Pomace	Juice and jam productions	Water	UAE	Solid–liquid ratio 25:1 mg/L, 750 W, 20 kHz, 10 min, 40% US amplitude	Cyanidin-, Delphinidin-, Malvidin-, Peonidin-, Petunidin-derivatives	1.39 mg CGE/g dw	[31]
*Solanum melongena* L. var. *serpentinum*	Eggplant	Peels	Canning factory	Ethanol	MAE	Solid–liquid ratio 1: 6.42 g/mL, EtOH 55.56%, 5.78 min, 298.84 W, pH 4.57	n.s.	8.54 mg CGE/L	[32]
*Vaccinium angustifolium* Aiton	Blueberry	Pomace	Berry processing	Ethanol	UAE	Solid–liquid ratio 1:20 g/mL, EtOH 50%, 40 °C, 90 min, 64 W, 35 kHz, pH 3.3	Cyanidin, Delphinidin, Malvidin, Petunidin	n.s.	[33]
*Vaccinium macrocarpon* Aiton	Cranberry	Pomace	Juice production	Water	SFE	2 g of sample, CO_2_:H_2_O = 50:50 (%, *w*/*w*), 50 °C, 4 h, flow rate 0.915 mL/min, 1 L CO_2_/min, 40 Mpa	Cyanidin-, Malvidin-, Peonidin-derivatives	2.45 mg CGE/g dw	[34]
*Vaccinium myrtillus* L.	Bilberry	Pomace	Juice production	Ethanol	HVED	Solid–liquid ratio 1:50 g/mL, EtOH 50%, HCl 1%, 25 °C, 15 min, 30 kV, 100 Hz	Cyanidin-, Delphinidin-, Malvidin-, Peonidin-, Petunidin-derivatives	1.09 mg/g dw	[35]
					PEF	Solid–liquid ratio 1:50 (g/mL), EtOH 50%, HCl 1%, 20 kV/cm, 100 pulse	Cyanidin-, Delphinidin-, Malvidin-, Peonidin-, Petunidin-derivatives	1.62 mg/g dw	[35]
					UAE	Solid–liquid ratio 1:50 (g/mL), EtOH 50% + HCl 1%, 80 °C, 15 min, 35 kHz	Cyanidin-, Delphinidin-, Malvidin-, Peonidin-, Petunidin-derivatives	0.95 mg/g dw	[35]
		Cake	Juice production	Water	UAE	solid–liquid ratio 5:1 g/L, 20 °C < T < 40 °C, 60 min, 16.7 W/cm^2^ stirring 300 rpm	Cyanidin-, Delphinidin-, Malvidin-, Peonidin-, Petunidin-derivatives	5.34 mg/g dw	[36]
*Vaccinum* spp.	Blueberry	Pomace	Juice production	NaDES	UAE	Solid–liquid ratio 1:60 g/mL, [(ChCl:OA = 1:1):water = 70:30] (*w*/*w*), 76 °C, 3.2 min, 325 W, 20 kHz	Cyanidin-, Delphinidin-, Malvidin-, Petunidin-derivatives	24.27 ± 0.05 mg CGE/g dw	[37]
*Vitis vinifera* L.	Red grape	Cake	Wine making	Solvent free	MAE	400 g of sample, 20 min, 1 W/g, 2.45 GHz	Cyanidin-, Delphinidin-, Malvidin-, Peonidin-, Petunidin-derivatives	4.49 ± 0.01 mg MGE/g dw	[38]
		Lees	Win making and juice production	NaDES	UAE	Solid–liquid ratio 1:10 g/mL, [(ChCl:MA = 1:1): water = 64.6:35.4] (*v*/*v*), 35 °C, 30.6 min, 341.5 W, 37 kHz	Delphinidin-, Malvidin-, Peonidin-, Petunidin-derivatives	6.55 mg MGE/g dw	[39]
		Pomace	Wine making	Ethanol	SFE	35 g of sample, EtOH 20%, 55 °C, 3 h, 25 g CO_2_/min, 100 bar	Delphinidin-, Malvidin-, Peonidin-, Petunidin-derivatives	0.30 ± 0.1 mg MGE/g dw (Petit Verdot) 3.8 ± 0.1 MGE/g dw (Tintilla) 3.20 ± 0.3 MGE/g dw (Syrah) 0.10 ± 0.1 MGE/g dw (Cabernet) 0.20 ± 0.1 MGE/g dw (Merlot) 2.00 ± 0.2 MGE/g dw (Tempranillo)	[40]
					PLE	EtOH 50%, 120 °C, 90 min, flow rate 5 g/min, 90 bar	Delphinidin-, Malvidin-, Peonidin-, Petunidin-derivatives	16.00 ± 1.0 mg MGE/g dw (Petit Verdot) 49.70 ± 2.8 mg MGE/g dw (Tintilla) 38.30 ± 0.6 mg MGE/g dw (Syrah) 11.10 ± 1.2 mg MGE/g dw (Cabernet) 10.10 ± 0.1 mg MGE/g (Merlot) 30.90 ± 1.0 mg MGE/g dw (Tempranillo)	[40]
					UAE	Solid–liquid ratio 1:4 g/mL, EtOH 44%, <50 °C, 3 min (15 s on–5 s off), 500 W, 20 KHz	n.s.	187.57 ± 4.69 mg/g	[41]
					Naviglio^®^ extractor	4 kg of sample, 12.2 kg EtOH 40%, 21 cycles, 1 min 25 s × cycle, total time 38 min (12 min in static phase, 26 min in dynamic phase)	Malvidin-, Peonidin-derivatives	4.00 g/L ± 0.05	[42]
			Wine making and juice or “pekmez”production	Glycerol	HAE	Solid–liquid ratio 1:22.4 g/mL, Glycerol 50% (*w*/*v*), 1000 rpm × 30 s, 15,000 rpm × 30 s	Delphinidin-, Malvidin-, Peonidin-, Petunidin-derivatives	1.39 mg CGE/g dw	[43]
			Wine making	NaDES	PLE	2 g of sample + 1 g of diatomaceous earth, [(ChCl:OA = 1:1):water = 30:60] (*w*/*w*), 60 °C, 10 min, 2 cycles, 1500 psi	Malvidin-, Peonidin-, Petunidin-derivatives	11.23 ± 1.36 mg/L	[44]
					UMAE	Solid–liquid ratio 0.3 g/mL, [(ChCl:CA = 2:1):water = 70:30] (*v*/*v*), UAE:50 W, 40 kHz, 10 min, MAE: 300 W, 10 min	Delphinidin-, Malvidin-, Peonidin-, Petunidin-derivatives	1.77 mg/g dw	[45]
					MAE	0.3 g of sample, [(ChCl:CA = 2:1):water = 75:25] (*v*/*v*), 100 W, 10 min	Delphinidin-, Malvidin-, Peonidin-, Petunidin-derivatives	~0.60 mg/g dw	[45]
					UAE	0.3 g of sample, [(ChCl:CA = 2:1):water = 75:25] (*v*/*v*), 50 W, 40 kHz, 10 min	Delphinidin-, Malvidin-, Peonidin-, Petunidin-derivatives	~0.30 mg/g dw	[45]
				Water	OHAE	2.5 g of sample, 5 mL NaCl 0.1 M, 13 s, 30 V/cm, 25 kHz, 25 mL water or CA 1% or LA 1%, stirring 30 min	Cyanidin-, Delphinidin-, Malvidin-, Peonidin-, Petunidin-derivatives	0.055 mg/g dw for water, 0.18 mg/g dw for CA 1% or LA 1%	[46]
					PLE	8 g sample, 2 g of dispersing agent, 120 °C, 1500 psi, 10 min, 2 cycles	n.s.	33.07 ± 1.14 mg/g	[41]
		Skin	Wine making	NaDES	UAE	Solid–liquid ratio 1.2:10 g/mL, [(CA:Maltose = 4:1):water = 76.20:23.8] (*w*/*w*), RT, 9.23 min	Cyanidin-, Delphinidin-, Malvidin-, Peonidin-derivatives	63.36 ± 1.51 mg CDGE/g dw	[47]
		Stem	Wine making	Ethanol	UAE	Solid–liquid ratio 1:4 g/mL, EtOH 44%, <50 °C, 3 min (15 s on–5 s off), 500 W, 20 KHz	n.s.	26.87 ± 2.00 mg/g	[41]
				Water	PLE	8 g sample, 2 g of dispersing agent, 120 °C, 1500 psi, 10 min, 2 cycles	n.s.	0.15 ± 0.01 mg/g	[41]

n.s.—not specified; CGE—cyanidin-3-*O*-glucoside equivalents; DGE—delphinidin 3-*O*-glucoside equivalent; MGE—malvidin-3-*O*-glucoside equivalents; CDGE—cyanidin-3-5-diglucoside equivalents; DGE—delphinidin-3-*O*-glucoside equivalents; dw—dry weight; LA—lactic acid; CA—citric acid; ChCl—choline chloride; Pro—propylene glycol; MA—malic acid; OA—oxalic acid; TMA—total monomeric anthocyanins; RT—room temperature.

## Supplementary Materials

The following supporting information can be downloaded at: https://www.mdpi.com/2076-3921/11/11/2169/s1, Figure S1: General anthocyanin structure; Table S1: Anthocyanin composition of the different plant sources considered in our survey with the substitute groups in the molecules, and analytical methods of detection used.

## Abbreviations

CA: citric acid; CD: cyclodextrin; CDGE: cyanidin-3-5-diglucoside equivalents; CGE: cyanidin-3-*O*-glucoside equivalents; ChCl: choline chloride; DGE: delphinidin 3-*O*-glucoside equivalent; dw: dry weight; EAE: enzyme-assisted extraction; GRAS: generally recognized as safe; h: hours; HAE: homogenizer-assisted extraction; HBA: hydrogen bond acceptor; HBD: hydrogen bond donor; HIUS-AE: high intensity ultrasound-assisted extraction; HVED: high voltage electrical discharge; LA: lactic acid; MA: malic acid; MAE: microwave-assisted extraction; MGE: malvidin-3-*O*-glucoside equivalents; min: minutes; NaDES: natural deep eutectic solvents; OA: oxalic acid; OHAE: ohmic heating-assisted extraction; PEF: pulsed electric field; PLE: pressurized liquid extraction; Pro: propylene glycol; RSM: response surface methodology; RT: room temperature; SE: stirring extraction; SFE: supercritical fluid extraction; TAC: total anthocyanin content; TMA: total monomeric anthocyanins; UAE: ultrasound-assisted extraction; UMAE: ultrasound/microwave-assisted extraction.
