# Supplementary material for: The Greening of Anthocyanins: Eco-Friendly Techniques for Their Recovery from Agri-Food By-Products
Source: Antioxidants (Basel). 2022 Nov 1;11(11):2169. doi: 10.3390/antiox11112169 (PMC9717736; doi:10.3390/antiox11112169)
Supplement: Supplementary file 1 [file antioxidants-11-02169-s001.zip › antioxidants-1974225-Table S1.pdf]

Table S1. Anthocyanin composition of the different plant sources considered in our survey with the substitute groups in the molecules, and analytical methods of detection used.

Plant species	Common name	By-product	Anthocyanin	R_1	R_2	R_3	R_4	Method of detection	Reference
Allium cepa L.	Red onion	Solid waste	Cyanidin-3-*O*-(6''-*O*-malonyl)glucoside	OH	H	(malonyl)glucoside	OH	LC–MS	17
			Cyanidin-3-*O*-glucoside	OH	H	Glucoside	OH	LC–MS	17
Amelanchier alnifolia Nutt. ex M.Roem.	Saskatoon	Pomace	Cyanidin-3-*O*-arabinoside	OH	H	Arabinoside	OH	HPLC-UV	18
			Cyanidin-3-O-galactoside	OH	H	Galactoside	OH	HPLC-UV	18
			Cyanidin-3-*O*-glucoside	OH	H	Glucoside	OH	HPLC-UV	18
			Delphinidin-3-*O*-arabinoside	OH	OH	Arabinoside	OH	HPLC-UV	18
			Delphinidin-3-*O*-glucoside	OH	OH	Glucoside	OH	HPLC-UV	18
Crocus sativus L.	Saffron	Tepals	Delphinidin-3-*O*-diglucoside	OH	OH	Diglucoside	OH	UPLC-MS	19
			Delphinidin-3-*O*-glucoside	OH	OH	Glucoside	OH	UPLC-MS	19
								LC-MS	22
								LC–MS	20
			Delphinidin-3,5-*O*-diglucoside	OH	OH	Glucoside	Glucoside	UPLC-MS	19

								LC-MS	22
								LC–MS	20
			Malvidin-3-*O*-diglucoside	OCH_3	OCH_3	Diglucoside	OH	UPLC-MS	19
			Petunidin-3-*O*-glucoside	OCH_3	OH	Glucoside	OH	LC–MS	20
								UPLC-MS	19
			Petunidin-3,5-*O*-diglucoside	OCH_3	OH	Glucoside	Glucoside	LC-MS	22
								LC–MS	20
			Cyanidin-3-*O*-(6''-*O*-caffeoyl)diglucoside	OH	H	(caffeoyl)diglucoside	OH	HPLC-MS	24
			Cyanidin-3-*O*-(6''-*O*-*cis*-*p*-coumaroyl)diglucoside	OH	H	(*cis*-*p*-coumaroyl) diglucoside	OH	HPLC-MS	24
Nitraria tangutorun Bobrov.	-	Seed meal	Cyanidin-3-*O*-(6''-*O*-*trans*-*p*-coumaroyl)diglucoside	OH	H	(*trans*-*p*-coumaroyl)diglucoside	OH	HPLC-MS	24
			Cyanidin-3-*O*-diglucoside	OH	H	Diglucoside	OH	HPLC-MS	24
			Delphinidin-3-*O*-(6''-*O*-caffeoyl)diglucoside	OH	OH	(caffeoyl) diglucoside	OH	HPLC-MS	24
			Pelargonidin-3-*O*-(6''-*O*-*p*-coumaroyl)diglucoside	H	H	(*p*-coumaroyl) diglucoside	OH	HPLC-MS	24

			Pelargonidin-3-*O*-diglucoside	H	H	Diglucoside	OH	HPLC-MS	24
Phaseolus vulgaris L.	Black bean	Hulls	Cyanidin-3-*O*-glucoside	OH	H	Glucoside	OH	HPLC-DAD	25
			Delphinidin-3-*O*-glucoside	OH	OH	Glucoside	OH	HPLC-DAD	25
			Malvidin-3-*O*-glucoside	OCH_3	OCH_3	Glucoside	OH	HPLC-DAD	25
			Malvidin-3,5-*O*-diglucoside	OCH_3	OCH_3	Glucoside	Glucoside	HPLC-DAD	25
Plinia cauliflora (Mart.) Kausel	Jabuticaba	Peel	Cyanidin-3-*O*-glucoside	OH	H	Glucoside	OH	LC-MS	27
Punica granatum L.	Pomegranate	Male flowers	Cyanidin-3,5-*O*-diglucoside	OH	H	Glucoside	Glucoside	UPLC-MS	28
			Pelargonidin-3-*O*-glucoside	H	H	Glucoside	OH	UPLC-MS	28
			Pelargonidin-3,5-*O*-diglucoside	H	H	Glucoside	Glucoside	UPLC-MS	28
		Peel	Cyanidin-3-*O*-glucoside	OH	H	Glucoside	OH	UPLC-MS	28
			Cyanidin-3,5-*O*-diglucoside	OH	H	Glucoside	Glucoside	UPLC-MS	28
			Pelargonidin-3-*O*-glucoside	H	H	Glucoside	OH	UPLC-MS	28
			Pelargonidin-3,5-*O*-diglucoside	H	H	Glucoside	Glucoside	UPLC-MS	28

Vaccinium angustifolium Aiton	Blueberry	Pomace	Cyanidin	OH	H	OH	OH	HPLC-DAD	33
			Delphinidin	OH	OH	OH	OH	HPLC-DAD	33
			Malvidin	OCH_3	OCH_3	OH	OH	HPLC-DAD	33
			Petunidin	OCH_3	OH	OH	OH	HPLC-DAD	33
Vaccinium macrocarpon Aiton	Cranberry	Pomace	Cyanidin-3-*O*-arabinoside	OH	H	Arabinoside	OH	HPLC-MS	34
			Cyanidin-3-*O*-galactoside	OH	H	Galactoside	OH	HPLC-MS	34
			Malvidin-3-*O*-galactoside	OCH_3	OCH_3	Galactoside	OH	HPLC-MS	34
			Malvidin-3-*O*-glucoside	OCH_3	OCH_3	Glucoside	OH	HPLC-MS	34
			Peonidin-3-*O*-arabinoside	OCH_3	H	Arabinoside	OH	HPLC-MS	34
			Peonidin-3-*O*-galactoside	OCH_3	H	Galactoside	OH	HPLC-MS	34
			Peonidin-3-*O*-glucoside	OCH_3	H	Glucoside	OH	HPLC-MS	34
Vaccinium myrtillus L.	Bilberry	Pomace	Cyanidin-3-*O*-arabinoside	OH	H	Arabinoside	OH	LC-MS	35
			Delphinidin-3-*O*-arabinoside	OH	OH	Arabinoside	OH	LC-MS	35

			Delphinidin-3-*O*-glucoside	OH	OH	Glucoside	OH	LC-MS	35
			Malvidin-3-*O*-glucoside	OCH_3	OCH_3	Glucoside	OH	LC-MS	35
			Peonidin-3-*O*-arabinoside	OCH_3	H	Arabinoside	OH	LC-MS	35
			Peonidin-3-*O*-glucoside	OCH_3	H	Glucoside	OH	LC-MS	35
			Petunidin-3-*O*-glucoside	OCH_3	OH	Glucoside	OH	LC-MS	35
Vaccinum spp.	Blueberry	Pomace	Cyanidin-3-*O*-glucoside	OH	H	Glucoside	OH	HPLC-DAD	37
			Delphinidin-3-*O*-arabinoside	OH	OH	Arabinoside	OH	HPLC-DAD	37
			Delphinidin-3-*O*-galactoside	OH	OH	Galactoside	OH	HPLC-DAD	37
			Delphinidin-3-*O*-glucoside	OH	OH	Glucoside	OH	HPLC-DAD	37
			Malvidin-3-*O*-arabinoside	OCH_3	OCH_3	Arabonoside	OH	HPLC-DAD	37
			Malvidin-3-*O*-galactoside	OCH_3	OCH_3	Galactoside	OH	HPLC-DAD	37
			Malvidin-3-*O*-glucoside	OCH_3	OCH_3	Glucoside	OH	HPLC-DAD	37
			Petunidin-3-*O*-arabinoside	OCH_3	OH	Arabinoside	OH	HPLC-DAD	37

			Petunidin-3-*O*-galactoside	OCH_3	OH	Galactoside	OH	HPLC-DAD	37
			Petunidin-3-*O*-glucoside	OCH_3	OH	Glucoside	OH	HPLC-DAD	37
Vitis vinifera L.	Red grape	Cake	Cyanidin-3-*O*-glucoside	OH	H	Glucoside	OH	HPLC-DAD	38
			Delphinidin-3-*O*-glucoside	OH	OH	Glucoside	OH	HPLC-DAD	38
			Malvidin-3-*O*-(6''-*O*-*p*-coumaroyl)glucoside	OCH_3	OCH_3	(*p*-coumaroyl) glucoside	OH	HPLC-DAD	38
			Malvidin-3-*O*-glucoside	OCH_3	OCH_3	Glucoside	OH	HPLC-DAD	38
			Peonidin-3-*O*-glucoside	OCH_3	H	Glucoside	OH	HPLC-DAD	38
			Petunidin-3-*O*-glucoside	OCH_3	OH	Glucoside	OH	HPLC-DAD	38
		Lees	Delphinidin-3-*O*-glucoside	OH	OH	Glucoside	OH	HPLC-DAD	39
			Malvidin-3-*O*-(6''-*O*-*p*-coumaroyl)glucoside	OCH_3	OCH_3	(*p*-coumaroyl) glucoside	OH	HPLC-DAD	39
			Malvidin-3-*O*-glucoside	OCH_3	OCH_3	Glucoside	OH	HPLC-DAD	39
			Peonidin-3-*O*-(6''-*O*-*p*-coumaroyl)glucoside	OCH_3	H	(*p*-coumaroyl) glucoside	OH	HPLC-DAD	39
			Petunidin-3-*O*-glucoside	OCH_3	OH	Glucoside	OH	HPLC-DAD	39

	Cyanidin-3-*O*-glucoside	OH	H	Glucoside	OH	HPLC-DAD	46
	Delphinidin-3-*O*-glucoside	OH	OH	Glucoside	OH	HPLC-DAD	45
						HPLC-DAD	46
	Malvidin	OCH_3	OCH_3	OH	OH	LC-MS	42
	Malvidin-3-*O*-(6''-*O*-acetyl)glucoside	OCH_3	OCH_3	(acetyl)glucoside	OH	LC-MS	42
	Malvidin-3-*O*-(6''-*O*-caffeoyl)glucoside	OCH_3	OCH_3	(caffeoyl)glucoside	OH	HPLC-MS	44
Pomace	Malvidin-3-*O*-(6''-*O*-*p*-coumaroyl)glucoside	OCH_3	OCH_3	(*p*-coumaroyl) glucoside	OH	HPLC-MS	44
	Malvidin-3-*O*-glucoside	OCH_3	OCH_3	Glucoside	OH	HPLC-MS	44
						LC-MS	42
						HPLC-DAD	45
						HPLC-DAD	46
	Peonidin-3-*O*-(6''-*O*-acetyl)glucoside	OCH_3	H	Acetylglucoside	OH	HPLC-MS	44
	Peonidin-3-*O*-(6''-*O*-*p*-coumaroyl)glucoside	OCH_3	H	(*p*-coumaroyl) glucoside	OH	HPLC-MS	44

					HPLC-DAD	46
Peonidin-3-*O*-glucoside	OCH_3	H	Glucoside	OH	LC-MS	42
					HPLC-DAD	45
Petunidin-3-*O*-(6''-O-p-coumaroyl)glucoside	OCH_3	OH	(*p*-coumaroyl) glucoside	OH	HPLC-MS	44
					HPLC-MS	44
Petunidin-3-*O*-glucoside	OCH_3	OH	Glucoside	OH	HPLC-DAD	45
					HPLC-DAD	46
