# Supplementary figures and images for: The Greening of Anthocyanins: Eco-Friendly Techniques for Their Recovery from Agri-Food By-Products
Source: Antioxidants (Basel). 2022 Nov 1;11(11):2169. doi: 10.3390/antiox11112169 (PMC9717736; doi:10.3390/antiox11112169)

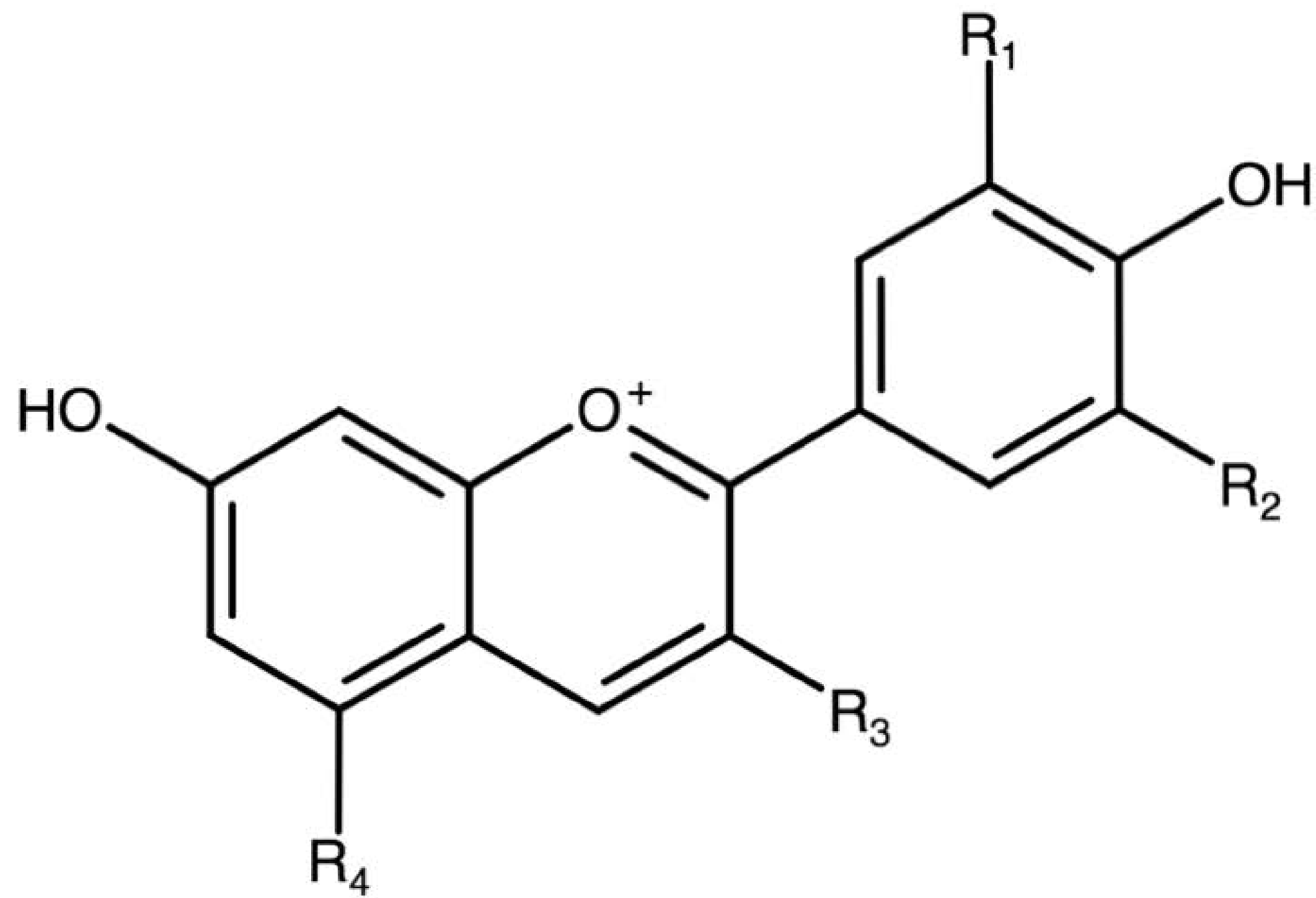

**Figure S1.** General anthocyanin structure.

Supplement: Supplementary file 1 [file antioxidants-11-02169-s001.zip › antioxidants-1974225-Figure S1.pdf]
